# The value of force and torque measurements in transanal total mesorectal excision (TaTME)

**DOI:** 10.1007/s10151-019-02057-z

**Published:** 2019-08-20

**Authors:** S. F. Hardon, R. J. van Kasteren, J. Dankelman, H. J. Bonjer, J. B. Tuynman, T. Horeman

**Affiliations:** 1 Department of Surgery, Amsterdam UMC–VU University Medical Center, P.O. Box 7057, 1007 MB Amsterdam, The Netherlands; 2 Department of BioMechanical Engineering, Delft University of Technology, Delft, The Netherlands

**Keywords:** Laparoscopy, Force assessment, Patient safety, Transanal surgery, TaTME

## Abstract

**Background:**

Transanal total mesorectal excision (TaTME) is associated with a relatively long learning curve. Force, motion, and time parameters are increasingly used for objective assessment of skills to enhance laparoscopic training efficacy. The aim of this study was to identify relevant metrics for accurate skill assessment in more complex transanal purse-string suturing.

**Methods:**

A box trainer was designed for TaTME and equipped with two custom made multi-DOF force/torque sensors. These sensors measured the applied forces in the axial direction of the instruments (Fz), instrument load orientation expressed in torque (Mx and My) on the entrance port, and the full tissue interaction force (Fft) at the intestine fixation point. In a construct validity study, novices for TaTME performed a purse-string suture to investigate which parameters can be used best to identify meaningful events during tissue manipulation and instrument handling.

**Results:**

Significant differences exist between pre- and post-training assessment for the mean axial force at the entrance port Fz (*p* = 0.01), mean torque in the entrance port Mx (*p* = 0.03) and mean force on the intestine during suturing Fft (*p* = 0.05). Furthermore, force levels during suturing exceed safety threshold values, potentially leading to dangerous complications such as rupture of the rectum.

**Conclusions:**

Forces and torque measured at the entrance port, and the tissue interaction force signatures provide detailed insight into instrument handling, instrument loading, and tissue handling during purse-string suturing in a TaTME training setup. This newly developed training setup for single-port laparoscopy that enables objective feedback has the potential to enhance surgical training in TaTME.

## Introduction

Transanal total mesorectal excision (TaTME) was introduced almost a decade ago to optimize the exposure in the distal narrow pelvis during the dissection [1–3]. Laparoscopic TME is associated with locoregional recurrence and survival rates similar to those for open surgery [4–7]. However, difficulties for the surgeon may arise if the patient has unfavourable characteristics such as narrow pelvic anatomy, male sex, or obesity [8–10]. Especially if the tumor is located in the low rectum, sphincter preservation procedures pose relatively high risks of leaving a positive circumferential resection margin (CRM) [11]. Due to these difficulties, following laparoscopic transanal minimally invasive surgery (TAMIS), the transanal TME (TaTME) technique has received tremendous attention all over the world and is being implemented at high speed [6, 12–14]. However, concerns exist about the learning curve and therefore, patient safety [15, 16]. Although cohort data reported proper short-term clinical outcomes, the real-world implementation so far has failed to better clinical outcomes [17]. The oncological outcome has yet to be published, preferably from randomized data [3, 18]. In the Netherlands, a national structured training pathway has been set up for TaTME, which acknowledges the need for training in new technical skills. Moreover, surgical anatomy is taught since the approach from different angles can potentially lead to intraoperative complications such as nerve or tissue damage, leading to anal sphincter dysfunction [10, 19].

A critical step in TaTME is placing the transanal purse-string suture before the full-thickness incision of the rectum. Apart from indicating the distal resection margin, the suture ensures the closing of the cut stump of the rectum preventing leaking of bowel fluids into the abdomen [20]. Various factors contribute to the quality of the purse-string. The number of stitches needed is said to relate to sufficient closure with a baseline of 9–12 stitches. Small gaps between sutures are critical, spiralling of the continuous suture has to be minimized, and having a correct angle of each stitch is found to be essential when closing the bowel [16]. Furthermore, poor purse-string technique poses a risk of tumor cell spillage and bacterial contamination [24]. Therefore, the necessity for appropriate training has been acknowledged early on in the development of the procedure [3, 16, 21–23]. Currently, an intensive international 2-day hands-on course followed by in-house proctoring is organized by our Department of Surgery [25].

However, there is a lack of assessment to ensure the level of competence before starting to use this technique in the operating room (OR). Both subjective and objective evaluation for fundamental laparoscopic skills and laparoscopic suturing have been extensively described, but not for this advanced single-port procedure [26–28]. Within this study, a force-measurement single incision laparoscopic surgery (SILS) training platform was developed, and construct validity was assessed.

Based on the consensus that the transanal placement of purse-string is a critical step within the procedure that should be well trained, the first goal of this study was to identify potential parameters for objective assessment of skills when performing a purse-string suture in TaTME training. A new custom made box trainer that measures the force and torque applied on the entrance port and the tissue interaction forces at the suture site was created to answer this question. The main aim was to determine learning effects with the proposed parameters in a study conducted with surgeons gaining experience in TaTME. The second goal was to investigate whether the recorded time, force, and torque data can be used to define metrics for assessment of technical skill for this relatively complex task.

## Materials and methods

### Trainer setup

**Fig. 1:**
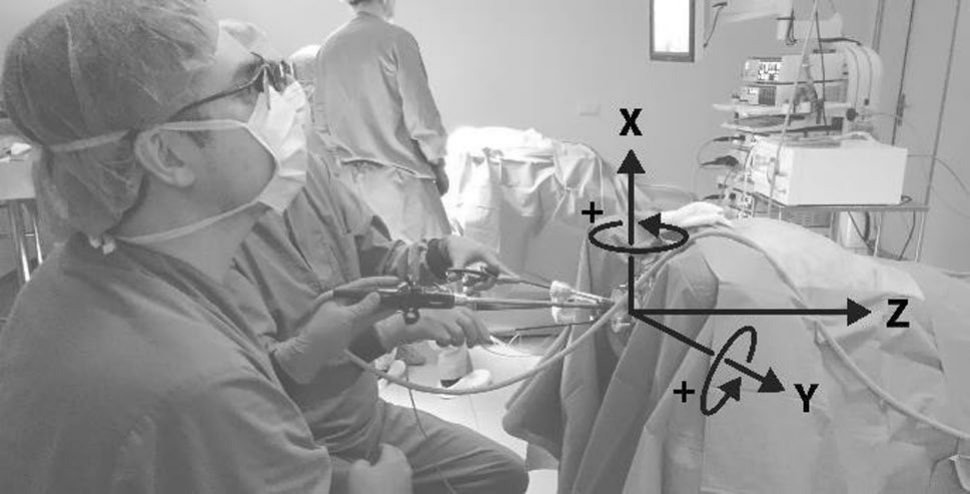
Surgeons training on the TaTME box trainer. An indication of the instrument configuration. Axis and rotations defined

The measurement system was designed to be compatible with the LapStar laparoscopic box trainer (Camtronics, Son, The Netherlands) (Fig. 1). This box trainer was adapted to allow the use of the GelPoint Path Transanal Acces Platform (Applied Medical, Rancho Santa Margarita, CA, USA), which is currently being used for the TaTME procedure in the OR, to approach reality in this simulation environment. Moreover, the setup allowed for insufflation to simulate pneumorectum. Observations during the TaTME course conducted in the OR show that the transanal purse-string runs around the wall of the bowel. Hence, the instruments rotate around the *x*- and *y*-axis of the port due to the used instrument configuration (Fig. 1). Torque and force are applied to deform the access platform or to overcome the friction between instruments and trocar valve for movement when the suture is placed.

### Measurement systems and software

**Fig. 2:**
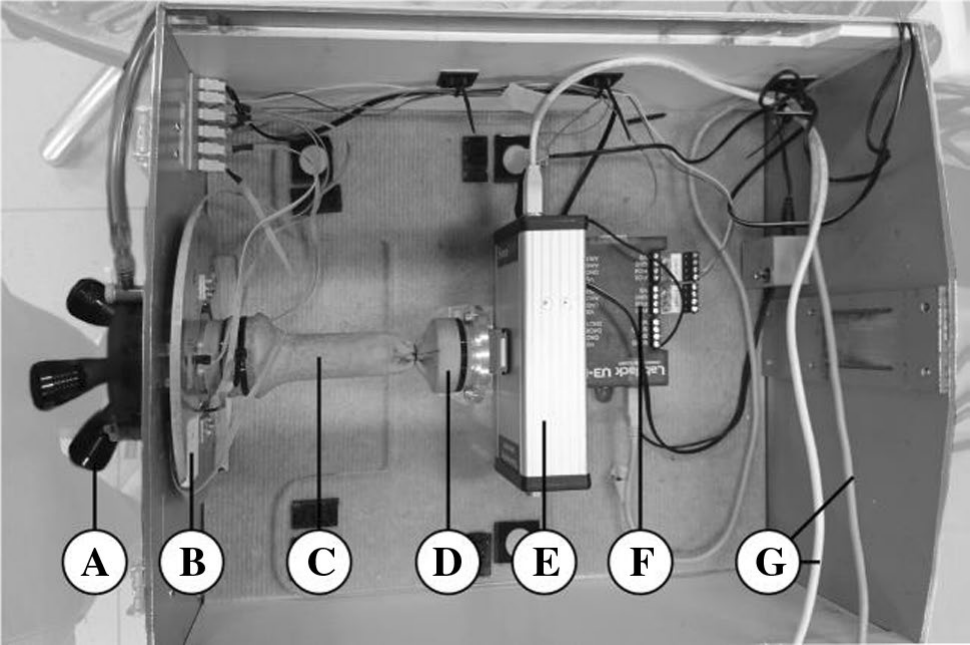
Box trainer components: (A) Gelpoint Path and insufflation tube. (B) Measurement port. (C) Dummy bowel. (D) Attachment to ForceTrap. (E) ForceSence Sensor, Medishield. (F) LabJack data acquisition device. (G) USB cables to laptop and tablet

**Fig. 3:**
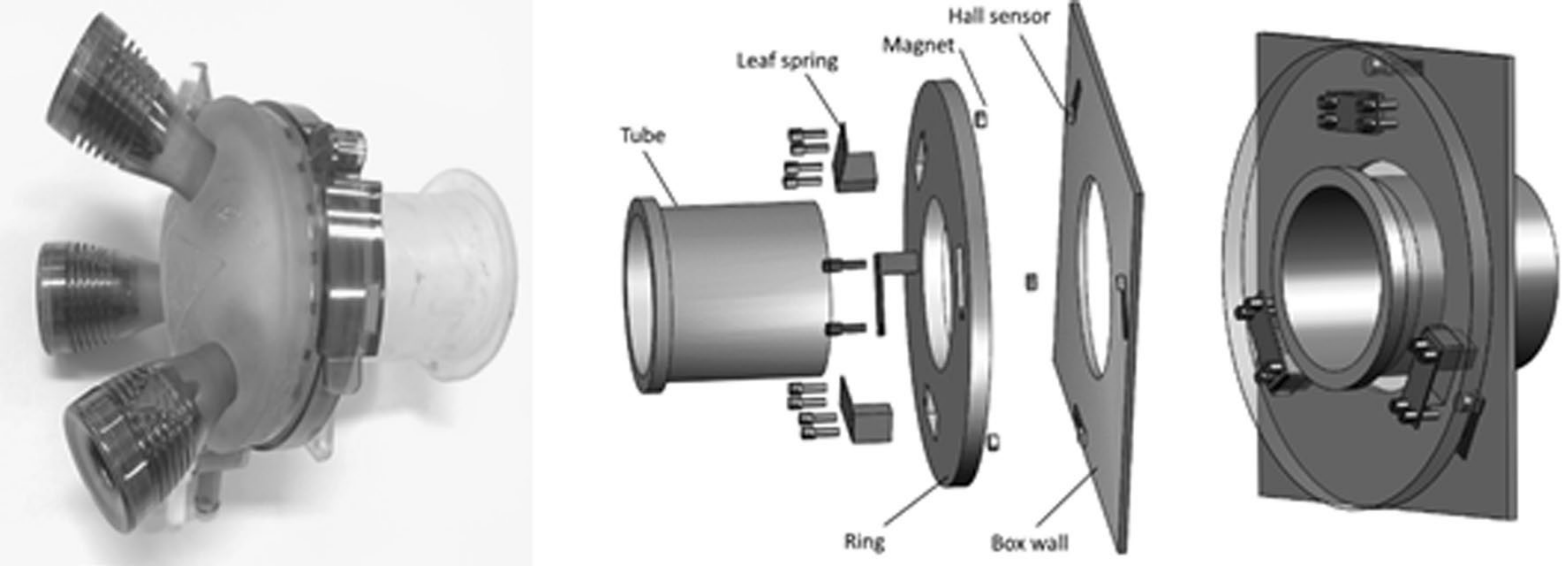
Port measurement setup. Left: Gelpoint Path. Middle: Exploded view of ring and leaf-spring component. Right: Configuration of the components

The box trainer, adapted for a single-port approach, was equipped with two measurement systems (Figs. 2, 3) to sense the force and torque at the access platform and the tissue interaction forces at the suture site. The platform was attached to the new measurement platform at the port site to measure instrument handling force and torque in the port. The distal part of the bowel was fixated to the port site to allow insufflation. The validated ForceSense measurement system (MediShield, Delft, The Netherlands) was installed to measure the tissue interaction forces during suturing [26, 29]. The proximal side of the bowel was fixated to the sensor through a rigid connection. Figure 3 shows a detailed depiction of the measurement port. The transanal access platform was fixated in a stiff aluminum tube that was rigidly connected to an inner ring. Three leaf spring constructions allow displacement measurements in the axial direction, and for measuring the rotation of the flat ring around the *x*- and *y*-axis. After calibration, the axial force Fz, the torques Mx, and My were calculated. Three Hall sensors, each accompanied by a magnet (∅3 mm, thickness 2 mm) were installed to measure the distance between the ring and box wall. The sensor outputs are measured using a LabJack U3 DAQ (Labjack Corporation, Lakewood, CO, USA) and written into text files with a sampling frequency of 50 Hz. After calibration, the specific force outputs for each hall sensor were calculated from the measured voltage outputs of the hall sensors. Tissue interaction force data were recorded with the sensor linked to a MS Surface tablet (Microsoft, Redmond, WA, USA) installed with ForceSense.net software. The ForceSense data were also recorded with a sampling frequency of 50 Hz. Structural components (Figs. 2, 3) were custom made at the 3 mE faculty of the Delft University of Technology. During the experiments, a calf’s colon was spanned between the ForceSense connector and Portal Sensor (Fig. 2c). Each participant used a new polydioxanone (PDS) 2.0 suture (Ethicon Inc., Somerville, NJ, USA) to ensure the same sharpness of the needle. The procedure was executed with conventional laparoscopic instruments (i.e., an atraumatic fenestrated grasper and a needle driver (Aesculap, B. Braun, Melsungen, Germany)) and a 30° laparoscope with a two-dimensional camera and imaging system (Olympus Corporation, Tokyo, Japan) for vision.

### Parameters

**Fig. 4:**
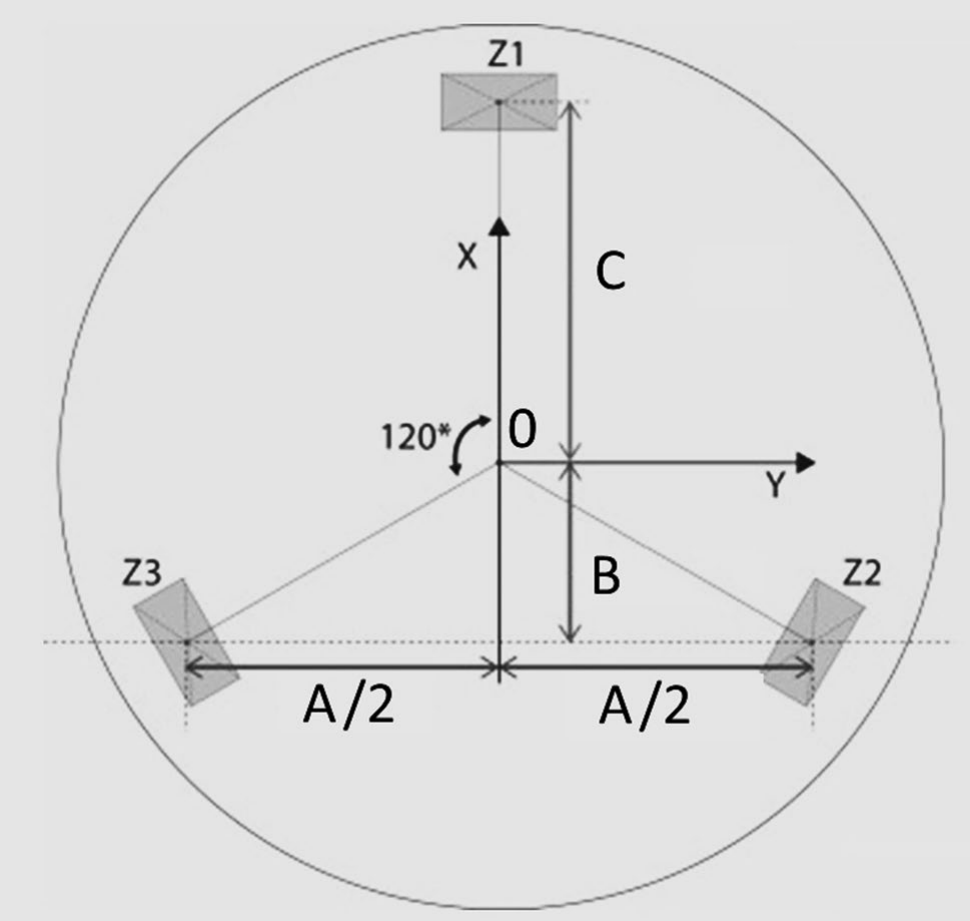
Force sensor configuration with dimensional aspects used to calculate the torque from the forces z1, z2, and z3

Before further processing, all raw data coming from the sensors was filtered with a 2nd order Butterworth filter with a cut-off frequency of 2.5 Hz [26]. The force and torque parameters measured in the port are shown in Table 1. Two characteristics were analysed per parameter: the maximum and mean output. For the torque representatives Mx and My it was expected that these parameters inform about instrument handling as they represent the instrument loading conditions that cause rotation of the instruments and, therefore, cause friction in the transanal platform. Mx and My are further specified as the mean torque generated clockwise (Mx neg, My neg) and anti-clockwise (Mx pos, My pos) to extract qualitative information about the configuration of the instrumentation in respect to the transanal access platform over time. The Fz parameter indicates whether the platform was pushed inwards or outwards. Having high readings on either Fz, Mx and My indicate risks on the loosening of the platform or rectal tissue damage. The suture was placed near the ForceSense sensor. Previous studies showed that the maximum needle/suture loading force before the rupture of the large intestine lies between 1 and 3 N [27, 30, 31]. From the comprehensive data, the mean and max force (Fft) on the intestine were recorded to relate performances to a risk of bowel leaks due to ruptured tissue at the suture site. The resultant force in the *z*-direction (Fz) in the port was calculated from the z1, z2, and z3 components in the port (Fig. 4) using Eq. 1. Figure 4 shows how the sensor locations, concerning the origin, were used to calculate the vertical and horizontal torque components (Mx and My) in the port using Eqs. 2 and 3.

**Table 1:** Parameters used to measure performance

Location	Parameter	Characteristic
Task time trial	Time	
Tissue interaction force	Fft	Max, mean
Axial Forces in port	Fz	Max, mean
Internal Torque in port	Mx	Max, mean
	Mx.pos	Mean
	Mx.neg	Mean
	My	Max, mean
	Mx.pos	Mean
	Mx.neg	Mean

1 $${\text{Fz}} [{\text{N}}] = {\text{Fz1}} + {\text{Fz2}} + {\text{Fz3}} .$$

2 $${\text{Mx}}\; [ {\text{Nm]}} = A/ 2({\text{Fz2}} - {\text{Fz3}}).$$

3 $${\text{My}}\; [ {\text{Nm]}} = B({\text{Fz2}} + {\text{Fz3}}) - (C \cdot {\text{Fz}}).$$

Table 2 shows that second-degree polynomial trend lines were fitted to express the voltage output in force (Fz1–Fz3) for each of the three force sensors (z1–z3). The functions and constants were obtained using MS Excel.

**Table 2:** Calibrations and root square for each of the three force sensors

Function	Measurement range (*N*)	Fit (*R*^2^)
Fz1 = 125.9 − 1.07 · 10^−3^√(*V* ∗ 8.37 · 10^9^ − 1.05 · 10^10^)	2.5 ≤ *x* ≤ 4.0	0.97
Fz2 = 39.8 − 3.48 · 10^−4^√(*V* ∗ 3.32 · 10^10^ − 8.10 · 10^10^)	2.5 ≤ *x* ≤ 3.2	0.93
Fz3 = 15.16 − 4.88 · 10^−5^√(*V* ∗ 4.09 · 10^11^ − 1.09 · 10^12^)	2.5 ≤ *x* ≤ 4.1	0.98

### Participants

Participants with experience in laparoscopic surgery were recruited during the TaTME course on 31 January and 1 February 2018. Subjects were selected based on their experience with a transanal purse-string suture. The trainees with experience of 0–10 purse-string sutures were considered novices for this procedure and were included in the study. Furthermore, the experience of the laparoscopist was considered. This information was obtained through a questionnaire before the training.

### Task

A full transanal purse-string suture consists of running the suture 360° along the wall of the bowel, with the last stitch overlapping the first to ensure full circumference of the intestine. Then the suture is pulled tight, closing the lumen, and tied with a surgical knot. Before participation, each participant was clearly instructed before starting the measurements and specifically asked to start the suture at the bottom (6 o’ clock) and work clockwise. For this study, participants were instructed not to tighten the suture and not to tie a knot, to ensure that the knot-tying phase does not influence the force measurements and data was interpreted correctly. Both measurement systems were started before the participant initiated the suture. A timestamp was given to both measurement systems at the first stitch to allow synchronization of the data afterward.

### Data analysis

Data files were obtained from the two different programs; the ForceTRAP’s online environment ForceSense.net for tissue interaction data and LJStreamUD software for port force and torque data. The data was off-line imported in MS excel for visualization and manual synchronization. Statistical analysis was conducted in SPSS Statistics V24.0 (IBM Corp., Armonk, NY, USA). First, a Kolmogorov–Smirnov test for normality was conducted on each performance parameter of the pre- and post-course measurement. Second, a paired samples *t* test was performed to compare pre- and post-course outcomes for each performance parameter of the novice group.

## Results

**Fig. 5:**
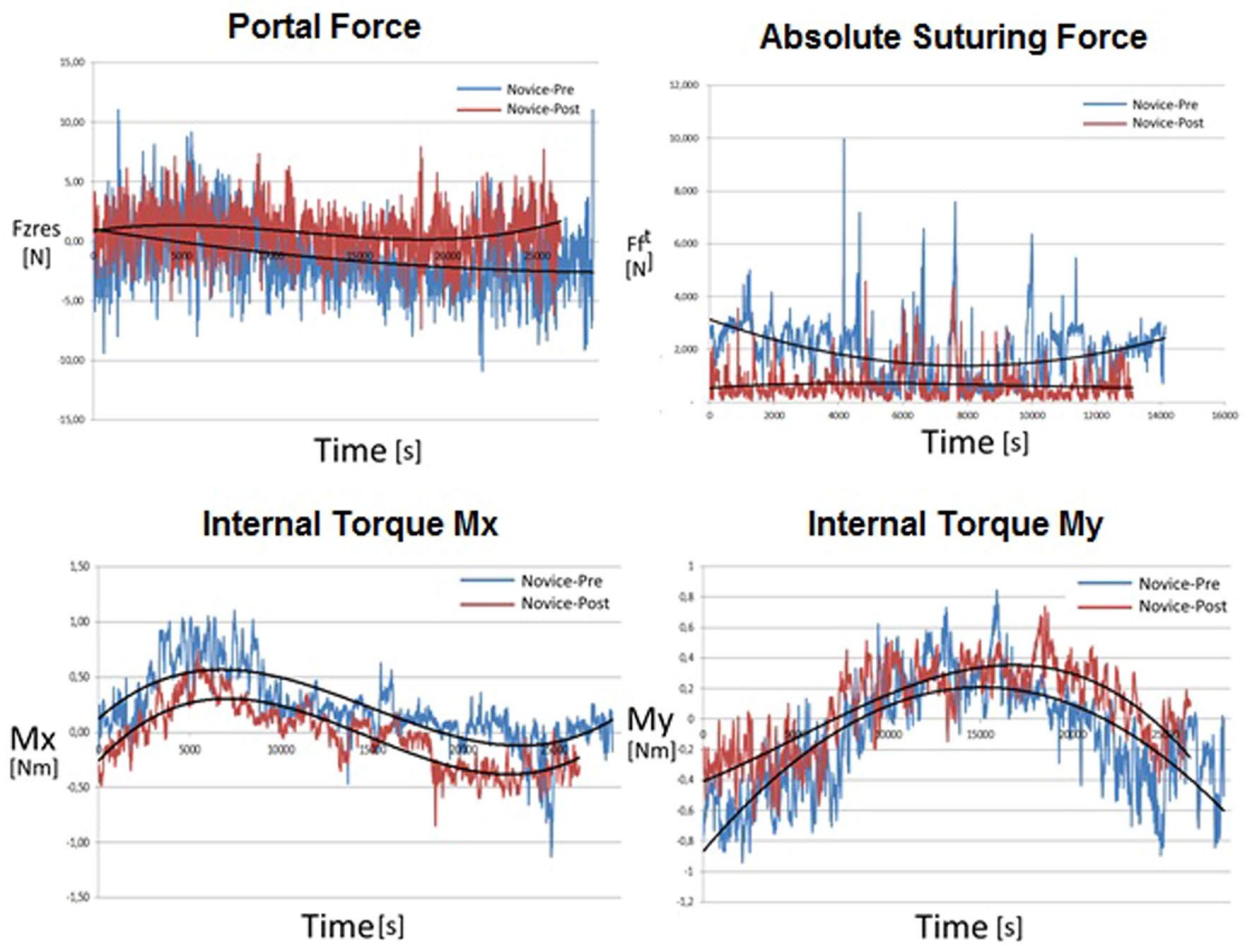
Example of a pre- and post-measurement for portal force, absolute portal force and torques Mx and My

**Fig. 6:**
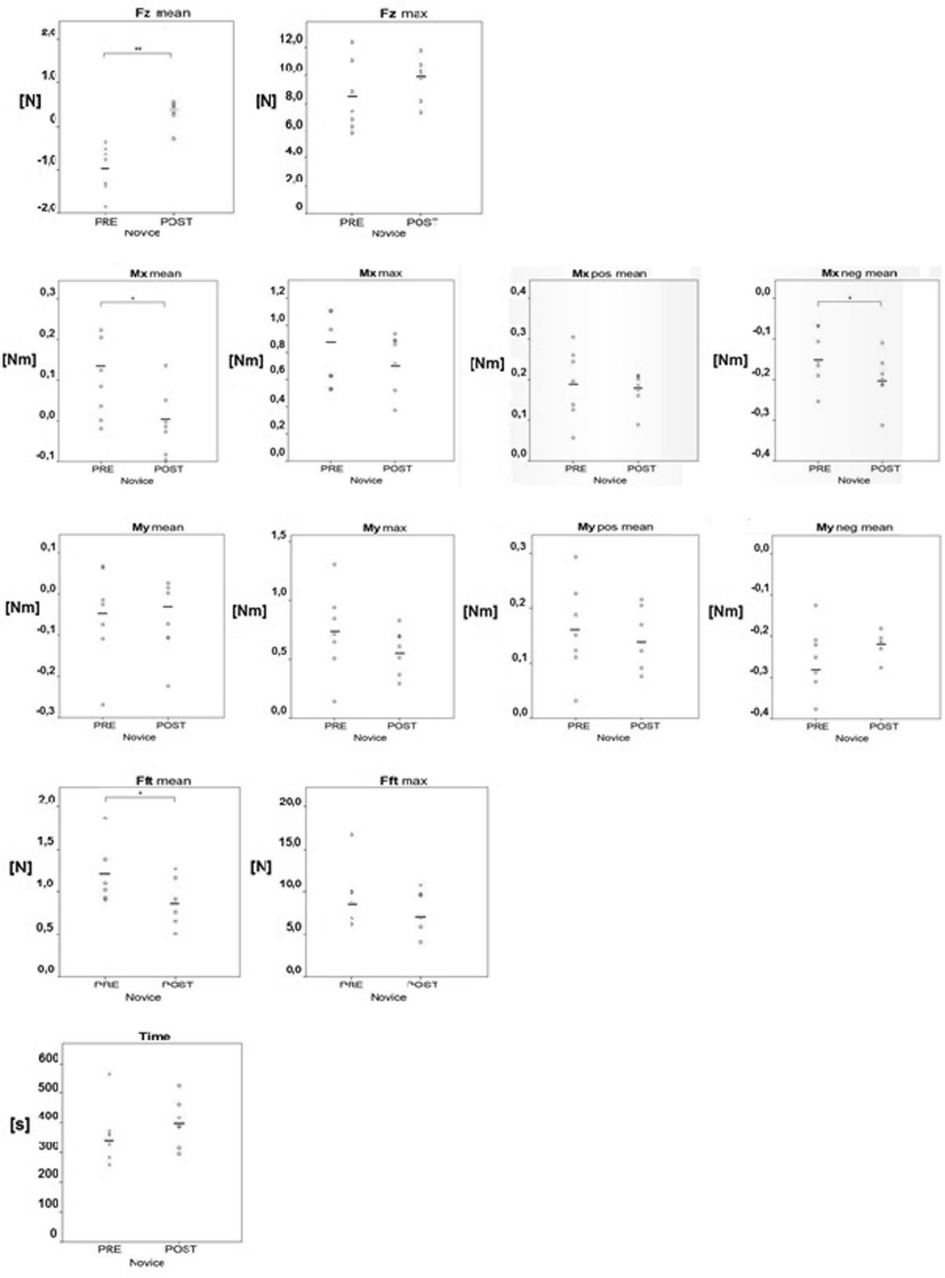
Portal Force parameters Fz mean and Fz max. Portal Torque parameters Mx mean, Mx max, Mx pos mean and Mx neg mean. Portal Torque parameters My mean, My max, My pos mean and My neg mean. Tissue Force parameters Fft mean and Fft max. Time to complete the task. *p < 0.05, **p < 0.01 indicates a statistical difference

Based on the questionnaire, a novices test group was formed with seven participants, all right-handed males. All participants were able to perform the pre- and post-course trials successfully accompanied by an expert laparoscopist. Figure 5 shows an example of the portal force, ForceTRAP Force, and torque graphs of novices at the beginning and end of the training course. A third-order polynomial was added to identify the essential signature of the curves. Figure 6 shows the scatter plots for the pre- and post-course trials for all parameters.

The signatures do show specific sinus like patterns corresponding with the suture location on the circular intestine. The patterns shift towards lower values during training as an indication for better symmetric behaviour and improved teamwork.

Kolmogorov–Smirnov tests for normality showed that the data for the task time was not normally distributed for the novice pre-training group, and the rest of the data was normally distributed. The paired samples *t* test comparing novices pre- and post-training outcomes showed significant differences for the mean Fz (1.32 N, *p* = 0.01), mean Mx (0.12 Nm, *p* = 0.03), mean Mx neg (0.037 Nm, *p* = 0.01) and mean absolute tissue interaction force Fft (0.21 N, *p* = 0.05).

It was observed that all participants exceeded the 3 N safety threshold for tissue manipulation force during placement of the transanal purse-string suture at the pre- and post-course assessment.

## Discussion

This study shows that force and torque measured at the entrance port, and the tissue interaction force signatures give risk-related insight in instrument handling, instrument loading, and tissue handling during purse-string suturing in a TaTME training setup. This newly developed laparoscopic single-port force measurement system enables objective feedback has the potential to enhance surgical training.

The most important results observed between the pre- and post-measurement are the differences in the mean force applied to the port in the *z*-direction and the torque generated around the sensor’s *x*-axis. Both results indicate that students learn to apply less inwards pressure and to keep the instrument shafts away more parallel to the neutral line in the horizontal plane. Furthermore, the lack of difference in task time is an observation that does not correspond to earlier studies conducted with conventional configurations.

### Portal force and torque

**Fig. 7:**
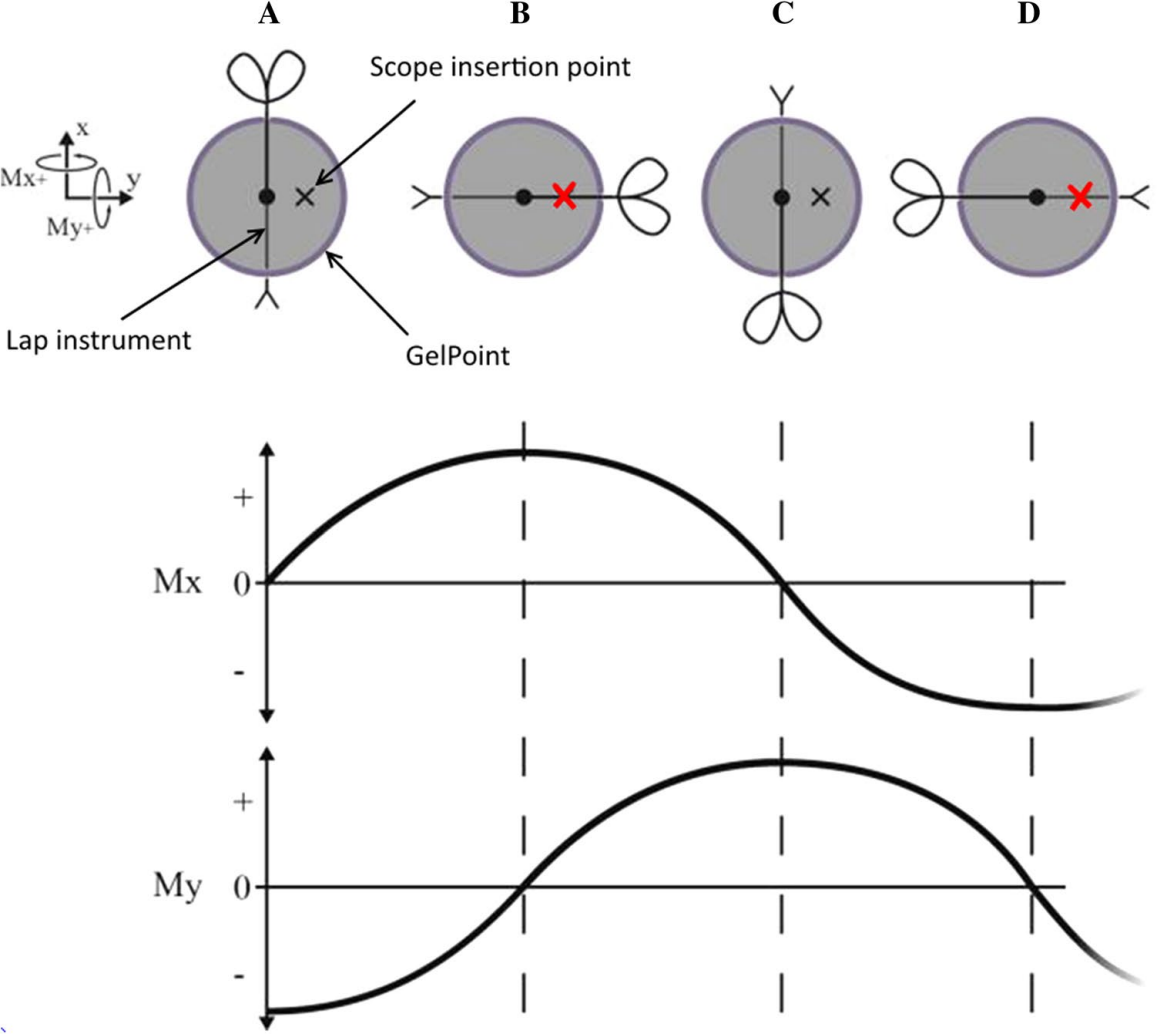
Mx and My torque graphs divided into four phases. The four top figures indicate the configuration of the instrument and laparoscope in the GelPoint Path during the suture. The red crosses indicate the two phases with an increased risk on instrument collision

Because of the 360° motion that is made when performing the transanal purse-string it is expected that the instrument shaft direction and thus internal torque in the port shifts during the circular suture. As participants were asked to start at the lowest point and to perform the suture clockwise, the torque direction follows a harmonic pathway as shown in Fig. 7. Those distinctive shapes can be seen in the raw data of Fig. 5 and can be used to assess the consistency and direction of the torque pattern. For example, when a conflict or collision occurs with the laparoscopist or laparoscope, a sudden change of pattern could indicate a non-smooth transition from one side to the other as observed many times during our experiment. The data suggest that the subjects improve most on the Mx parameter outcome. Mx is the torque generated due to instrument movements around the vertical axis in the horizontal plane and can be used to visualize conflicts between the surgeon’s instruments with the scope and arms of the laparoscopist. Therefore, improvements in this Mx parameter outcome indicate an improved collaboration due to fewer struggles to move instruments from right to left and vice versa.

**Fig. 8:**
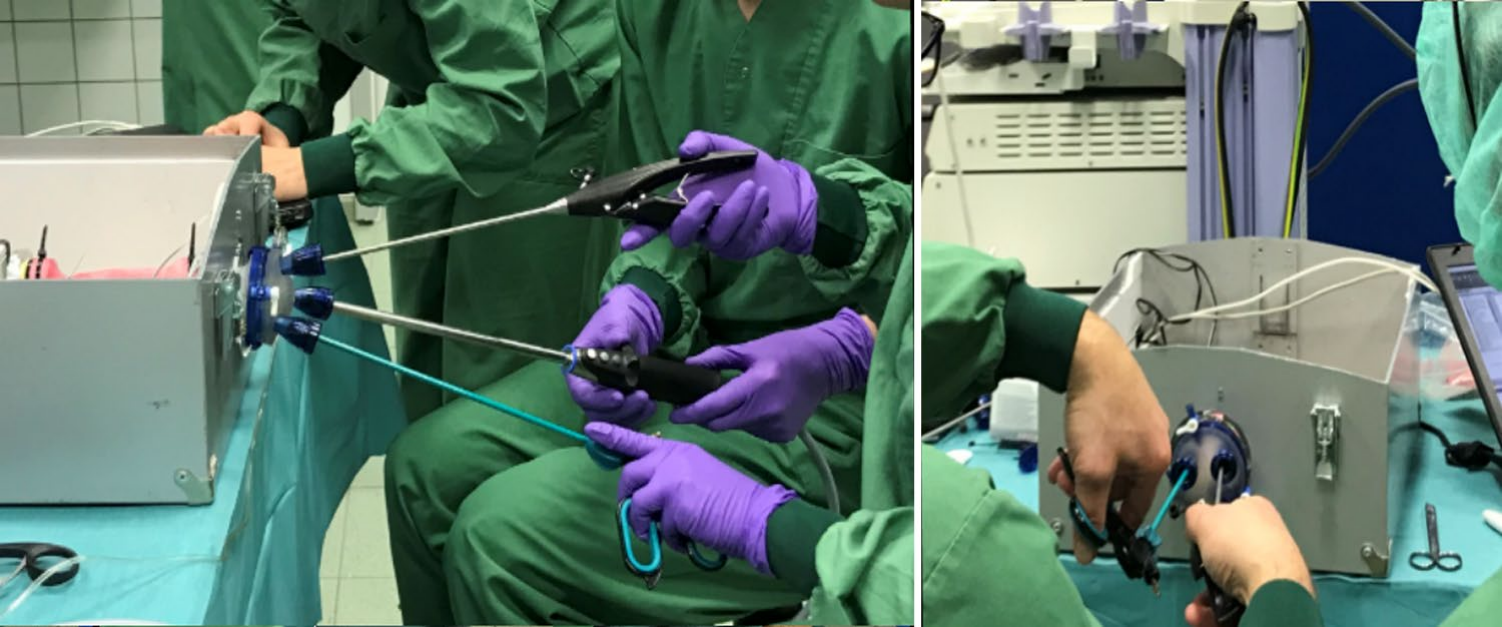
The pre-training picture (left) indicates a large angle between the two instruments and less wrist articulation. The post-training picture (right) shows more parallel instrument handling between instruments facilitated by more active wrist articulation

Despite the small study population, the data strongly indicates that absolute forces and internal torques are reduced during the training session. This correlates with the observation that students first use their arms to generate the required instrument movements while at the end of the training session the participants keep their hands closer together, use wrist movements and keep their instruments more parallel to the neutral line of the intestine (Fig. 8). In some cases, the raw torque patterns of the internal torque are shifted away from the 0-line. Although not investigated, it is likely that the arms of the surgeon are not perfectly aligned with the neutral line of the port and intestine due to a non-optimal height of the surgeon’s chair or table. For real surgery, this shows how important it is to adjust the patient and chair to the correct height before surgery starts. In literature, anal dysfunction after surgery is only linked to operating time [32–34]. It can be of interest to relate clinical outcome to portal force and torque during instrument handling.

Besides being a valid metric for skills assessment in surgical tasks, force information in a training setting provides information about the risk of tissue damage due to excessive interaction force [26, 35, 36]. In both pre- and post-course assessments in this study, it was observed that all participants exceeded the safety thresholds for tissue damage. Previous research of Horeman et al. [31] showed that tissue damage occurs relatively quickly in porcine intestines when pulling a suture. Considering these outcomes and the results of our present study, the repetitive practicing of this surgical technique in a safe simulation environment outside the OR is highly recommended. Moreover, trainees may benefit from deliberate practice if qualitative and quantitative force-feedback is provided during training.

Previous studies show that after training in laparoscopic skills with virtual reality or box trainers, a significant reduction in task time is observed [26, 35–39]. Therefore, task time is often used as a performance parameter to indicate efficient learning. Within our pilot study, a reduction in task time is not observed, and the average task time in the novice post measurements even seem to increase. In contrast to this, the natural gradient of force parameter learning curves is more of a straight line without specific force-feedback and, therefore, indicates a limited decrease of tissue manipulation skills [40, 41]. However, the averaged force parameters outcomes in this study do show a significant decrease in force parameters between pre- and post-course assessment.

Therefore, the discriminating power of time and force parameters is likely to depend on the complexity of the assessed task. As the TaTME procedure is challenging due to its three-dimensional complexity and narrow workspace, surgeons recognize the consequence of rupturing the tissue as a suture needs to be replaced, or the bowel starts to leak. Therefore, the nature of this task makes the surgeons a lot more aware of their actions during training. As a result, surgeons try to apply the learned techniques properly with a focus on correct tissue manipulation and instrument handling and less on speed.

Due to the distinctive sinusoid Mx and My patterns seen in Fig. 7, it may be interesting to look at performance parameters that correlate the fit between the trainee’s force pattern and the theoretical force pattern. Consequently, unsymmetrical behaviour around the zero-force line or differences in amplitude/frequency ratio can be easily used as a measure for someone’s instrument alignment and performance consistency. Further research with a larger study population is needed to link those FPA parameters to specific flaws in natural operator-surgical site alignment or instrument use. Moreover, insight into the parameter learning curves and how they deviate from learning curves obtained during studies conducted with a conventional multiport laparoscopic approach can be gained.

### Limitations

Unfortunately, the power in this study is too low to observe statistically significant results for the maximum force parameters generated on the port and suture that specifically indicate a decreased risk of tissue damage. However, a power analysis suggests that, respectively, 90, 27 and 27 participants are needed to show significant differences (i.e., Power of 1-beta = 0.8) between the Task time, Fz max and Fft Max parameters of the pre- and post-training group. Differently from previous learning effect studies on interaction force-related parameters [40, 41], we found a clear indication that the force levels are naturally decreasing during training without the use of force feedback. Tracking the learning curves of the used force and torque parameters should give insight on how subjects shift their focus on task time (i.e. how to finish as fast as possible) to instrument handling (i.e. body posture and interaction with laparoscopist) and tissue interaction force (i.e. how to lower the risk on tissue damage). Moreover, future studies should also focus on women and left-handed surgeons to better understand the role of the force and torque measurement system in a more diverse group.

## Conclusions

A novel force- and torque-based measurement system suitable for single-port box training was established. Tissue trauma-related force and torque metrics were identified and learning effects were indicted. This system has potential for feedback and assessment during preclinical skills acquisition for transanal surgical procedures.
